# Supplementary material for: Genetic Causal Association between Iron Status and Osteoarthritis: A Two-Sample Mendelian Randomization
Source: Nutrients. 2022 Sep 6;14(18):3683. doi: 10.3390/nu14183683 (PMC9501024; doi:10.3390/nu14183683)
Supplement: Supplementary file 1 [file nutrients-14-03683-s001.zip › nutrients-1831912-supplementary.pdf]

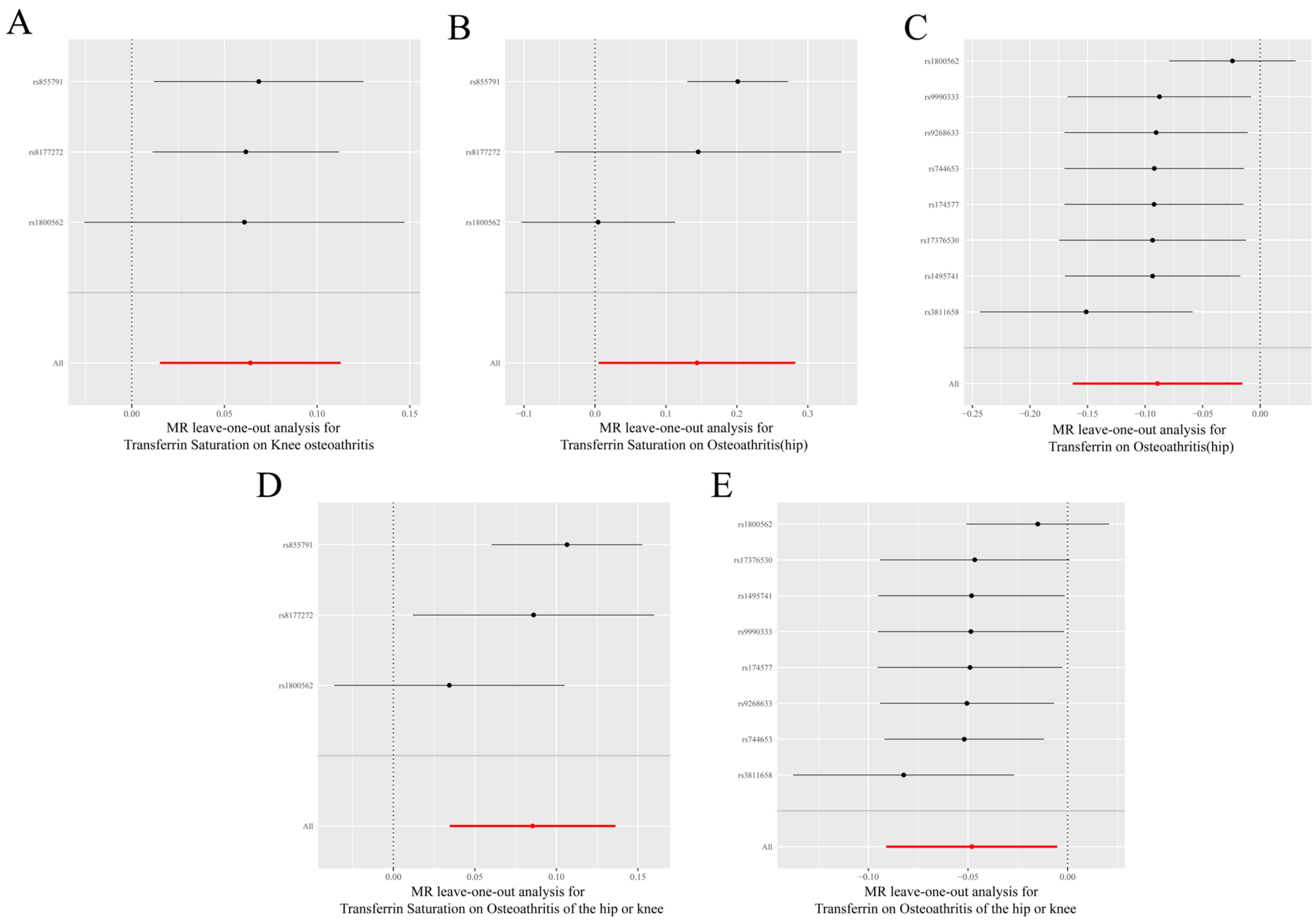

Supplemental Figure S1. MR results of leave-one-out sensitivity analysis for (**A**) Transferrin saturation and knee osteoarthritis (KOA); (**B**) Transferrin saturation and hip osteoarthritis (HOA); (**C**) Transferrin and HOA.; (**D**) Transferrin saturation and H/KOA.; (**E**) Transferrin and H/KOA.
